# Neither phylogenomic nor palaeontological data support a Palaeogene origin of placental mammals

**DOI:** 10.1098/rsbl.2013.1003

**Published:** 2014-01

**Authors:** Mario dos Reis, Philip C. J. Donoghue, Ziheng Yang

**Affiliations:** 1 Department of Genetics, Evolution and Environment, University College London, Gower St., London WC1E 6BT, UK; 2 School of Earth Sciences, University of Bristol, Wills Memorial Building, Queens Road, Bristol BS8 1RJ, UK

**Keywords:** placental mammal, fossil, divergence time

## Abstract

O'Leary *et al*. (O'Leary *et al.* 2013 *Science* **339**, 662–667. (doi:10.1126/science.1229237)) performed a fossil-only dating analysis of mammals, concluding that the ancestor of placentals post-dated the Cretaceous–Palaeogene boundary, contradicting previous palaeontological and molecular studies that placed the ancestor in the Cretaceous. They incorrectly used fossil ages as species divergence times for crown groups, while in fact the former should merely form minimum-age bounds for the latter. Statistical analyses of the fossil record have shown that crown groups are significantly older than the oldest ingroup fossil, so that fossils do not directly reflect the true ages of clades. Here, we analyse a 20 million nucleotide genome-scale alignment in conjunction with a probabilistic interpretation of the fossil ages from O'Leary *et al.* Our combined analysis of fossils and molecules demonstrates that Placentalia originated in the Cretaceous.

## 1. Introduction

Placental mammals (crown Eutheria) appear in the fossil record after the Cretaceous–Palaeogene (K–Pg) mass extinction event 66 Ma ago, when an estimated 76% of all species became extinct [1]. The sudden appearance of placental fossils in the Palaeogene is consistent with an adaptive radiation of mammals assuming ecological niches left vacant by dinosaurs. Molecular and palaeontological studies have supported a Cretaceous origin of Placentalia, but the age of placental mammal ordinal level crown groups (the ‘modern’ orders) relative to the K–Pg event has been the subject of protracted debate [2–7]. An explosive model of placental radiation, in which the last common ancestor of placentals post-dated the K–Pg event, has been rejected by molecular and palaeontological studies [2–6].

Recently, O'Leary *et al.* [8] (see also [9,10]) analysed a data matrix of 4541 morphological characters from 46 extant and 40 fossil mammal species to reconstruct and date the last common ancestor of placentals. They incorrectly estimate the age of living clades by the age of their oldest fossil representatives. Thus, for example, they translate the age of the oldest fossil placental, the ‘condylarth’ *Protungulatum donnae* (64.85 Ma), directly into the age of a phylogenetically remote placental ancestor, which they infer to have been an agile shrew-like organism that post-dated the K–Pg event. By contrast, recent molecular studies have incorporated both fossil and molecular evidence and have firmly placed the common ancestor of placentals in the Cretaceous, 117–88 Ma [2,3]. O'Leary *et al.* seek to reignite a controversy over the age of the placental ancestor that has otherwise been settled [2,3].

Concerned that the timescale of placental mammal evolution presented in O'Leary *et al.* [8] may become accepted uncritically, we highlight and remedy the serious shortcomings manifest in their study. The objectives of our paper are threefold: (i) to emphasize why fossil ages cannot be directly used as proxies for ages of clades, (ii) to show how stochastic models of the fossil record can be used to construct calibrations and date molecular trees and (iii) to analyse a phylogenomic dataset to demonstrate that Placentalia originated in the Cretaceous.

## 2. Material and methods

Using fossil ages as direct estimates of clade age is unjustified. However, clade age can be estimated based on statistical analysis of the fossil evidence. For example, Wilkinson *et al.* [11] used a stochastic model of fossil preservation and discovery to determine an 88.6–57.6 Ma age for crown Primates based on the age of the oldest crown primate (54.8 Ma) and allowing for the effects of the K–Pg extinction. This estimate, based on fossils alone, is 5–62% older than the age of the oldest fossil crown primate. Evidently, fossil ages do not directly represent clade ages and it is for this reason that in attempting to derive an evolutionary timescale, literal reading of the fossil record has given way to molecular clock methodology which uses statistical distributions to describe uncertainties in fossil calibrations.

Thus, we undertook a molecular clock study analysing the alignments of 14 632 orthologous genes (20.6 million nucleotides) of dos Reis *et al.* [3] to estimate the age of Placentalia. The program MCMCTree [12] was used to perform Bayesian estimation of divergence times using the approximate likelihood method [13]. We used the auto-correlated rates model to construct the prior of the rates. The time unit is 1 Myr. We used a gamma prior on the mean rate, *G*(1, 100), with mean 0.01 (meaning 1 substitution per site per 10^8^ years) and a gamma prior on the rate drift coefficient *σ*^2^, *G*(1, 100). The parameters for the birth–death process were set to *λ* = *μ* = 1 and *ρ* = 0. The alignment was divided into 20 partitions according to overall substitution rate, and only the first and second codon positions were used in the analysis [3]. The substitution model was HKY + *Γ*_4_.

We implemented two fossil calibration strategies. In strategy 1, we used the K–Pg-based primate calibration proposed by Wilkinson *et al.* [11] together with the same broad calibration (191.1–162.9 Ma) on the crown mammal root used by dos Reis *et al.* [3] (a calibration on the root is always necessary with MCMCTree). The calibration density derived in [11] is the posterior distribution of their fossil-only analysis, which is then used to construct the time prior in our molecular clock analysis. For strategy 2, we used the two calibrations from strategy 1 supplemented by 16 minimum-bound calibrations based on fossil ages from O'Leary *et al.* [8] (table 1). In both strategies, we treated all bounds as soft, i.e. the probability that the clade age is outside the calibration bounds is small, but non-zero. The calibration densities are combined with a birth–death process to construct the prior of times for all nodes in the phylogeny, providing a stochastic interpretation of the fossil information. The posterior estimates of times are then the result of combining the prior (the fossil information) with the likelihood of the data (the molecular sequence alignment). We compared the results of these analyses with the results of [3] which implemented a much greater suite of fossil calibrations.

**Table 1.** Ages of crown mammal groups.

crown group	oldest fossil according to O'Leary *et al.* [10]	fossil age (Ma)	95% CI of time posterior (Ma)					
			strategy 1		strategy 2		dos Reis *et al.* [3]	
Mammal	Dryolestida	166.4	162.4	188.8	184.7	193.2	174.5	191.8
Theria	*Sinodelphis szalayi*	127.5	140.5	168.5	179.7	191.5	170.4	181.7
Marsupialia	*Peradectes minor*	64.85	60.4	79.9	77.3	94.0	50.7	83.7
Placentalia	*Protungulatum donnae*	64.85	72.4	87.6	100.8	107.4	88.3	91.6
Afrotheria	*Prodiacodon crustulum*	64.85	56.2	68.3	78.6	84.5	68.5	72.4
Paenungulate	*Simpsonotus praecursor*	61.8	46.7	57.1	65.4	71.0	57.7	61.8
Xenartha	*Riosgotherium yanei*	58.3	52.3	63.9	73.3	79.0	67.3	72.4
Boreotheria	*P. donnae*	64.85	66.5	80.6	93.0	99.0	81.1	83.8
Laurasiatheria	*P. donnae*	64.85	60.6	73.4	84.9	90.3	74.8	77.1
Lipotyphla	*Litolestes ignotus*	58.3	48.3	58.9	67.9	72.9	60.6	61.8
Carnivora	*Hesperocyon gregarius*	43.3	39.4	48.2	55.1	60.2	52.0	55.9
Chiroptera	*Archaeonycteris praecursor*	55.5	47.0	57.2	65.9	70.7	57.6	60.8
Euarchontoglires	*Purgatorius coracis*	64.8	60.8	73.7	85.3	90.7	74.6	77.0
Glires	*Mimotona wana*	63.4	56.9	69.0	80.1	85.1	69.6	71.8
Lagomorpha	Leporidae	53	35.0	43.0	52.8	54.5	45.8	49.3
Rodentia	*Sciurus* sp.	56.8	51.4	62.5	72.8	77.2	63.4	65.5
Euarchonta	*P. coracis*	64.85	59.6	72.2	83.7	88.9	73.0	75.3
Primates	*Teilhardina brandti*	53.1	55.3	67.1	77.8	82.7	67.8	70.1

The trees with fossil calibrations are available as the electronic supplementary material. The genome-scale alignment is available at http://abacus.gene.ucl.ac.uk/ziheng/data.html.

## 3. Results and discussion

**Figure 1.**
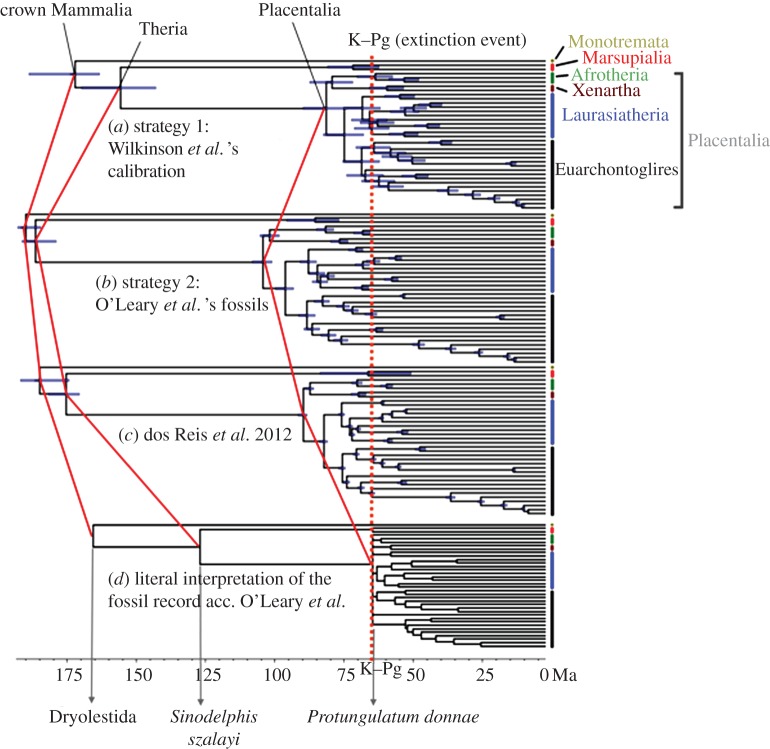
The age of Placentalia. (*a*) Phylogeny of 36 mammals (1 monotreme, 2 marsupials and 33 placentals), with divergence times estimated using calibration strategy 1. (*b*) Times estimated using strategy 2. (*c*) Time estimates from [3]. (*d*) Clade ages fixed to the fossil ages according to [8] (with the ages of intermediate nodes interpolated using the birth–death process by running MCMCTree without using molecular data). The tree topology with species names is given in the electronic supplementary material, figure S1.

**Figure 2.**
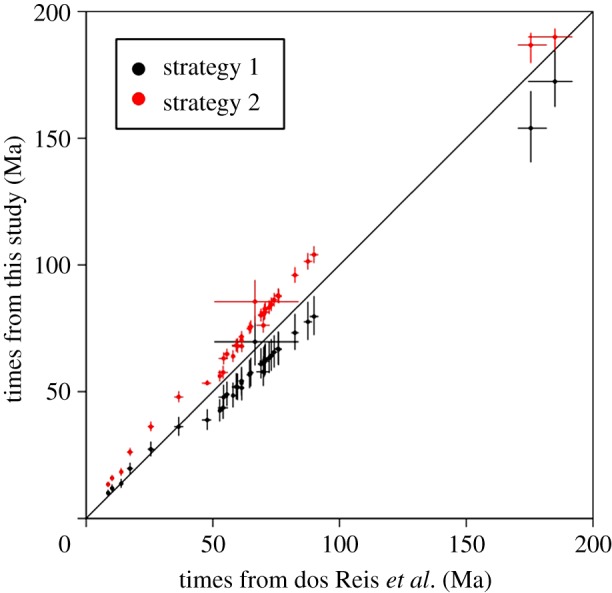
Comparisons of time estimates from this study (*y*-axis) versus those of dos Reis *et al.* [3] (*x*-axis). Dots indicate posterior mean times, and horizontal and vertical bars show the corresponding 95% CIs. The diagonal line is *y* = *x*.

The posterior age of Placentalia using calibration strategy 1 is 87.6–72.1 Ma (table 1 and figure 1*a*), and using calibration strategy 2 it is 108–100 Ma (table 1 and figure 1*b*). Thus, with the uncertainties in the calibrations accounted for, the estimated age of Placentalia under the two strategies is 108–72.1 Ma. This is 11.2–66.5% older than the 64.85 Ma age based on *P. donnae* (table 1 and figure 1*d*), the oldest placental fossil recognized in [8]. In general, strategy 1 produced younger age estimates for all nodes in the tree, with large uncertainties in the estimates, while strategy 2 produced more precise, but older estimates (table 1 and figure 2). The study by [3] produced time estimates that are intermediate between the two estimates in this study (table 1, and figures 1*c* and 2).

O'Leary *et al.* [8] recognize that ‘Ghost lineage estimates are minimum divergence dates and may underestimate the timing of actual splits’, but they subsequently abandoned this premise and accepted their fossil-based minimum-age constraints as though they were divergence times in inferring the biogeography and palaeoenvironment of a Palaeogene placental ancestor. Ghost lineage analysis was devised originally to estimate minimum gaps in the fossil record because sister clades are age-equivalent. However, in attempting to date a clade, this exercise is akin to estimating the age of a mother by using the age of her oldest child. Furthermore, even their age interpretation of the oldest placental (and laurasiatherian) fossil that they recognize, *P. donnae*, is questionable because there are putative records of *P. donnae* from the latest Cretaceous [14–16]. Indeed, the systematic interpretation of *P. donnae* [8] requires the pre-existence of the common ancestors that extant Laurasiatheria share with their successive placental sister-lineages, Epitheria and Boreotheria. Evidence in favour of a pre-Palaeogene history of crown placentals, implied by *P. donnae*, is confirmed here by the time estimates obtained using the molecular data and fossil calibrations (figure 1).

Although the three molecular clock analyses that exploit the same sequence alignment use different calibration strategies, the resulting divergence time estimates are highly correlated (table 1 and figure 2). The correlations between strategy 1 and 2, between strategy 1 and reference [3] and between strategy 2 and [3] are 99.4%, 99.5% and 99.6%, respectively. Thus, even allowing for the differences in which palaeontological data inform the divergence time analyses, we can conclude with confidence that the age of Placentalia is 1.18–1.20 times the age of Laurasiatheria. Thus, even if we followed O'Leary *et al*. [8] in their interpretation of their preferred oldest record of *P. donnae* as the absolute age of the crown Laurasiatheria (64.85 Ma), Placentalia must be 64.85 × 1.18 to 1.20 = 76.5–77.8 Ma. Yet, O'Leary assigns both Laurasiatheria and Placentalia the age of 64.85 Ma.

Despite the high correlations, reflecting the informativeness of the genome-scale sequence alignment, the three divergence time analyses differ in their absolute age estimates for Placentalia, as well as for many other intrinsic clades. These differences reflect differing degrees of temporal uncertainty inherent in the calibration strategies. Strategy 1 included only two fossil calibrations. Thus, the posterior time estimates for the uncalibrated nodes in the tree are sensitive to the birth–death and rate prior and exhibit high uncertainty. Ideally, divergence time analyses should incorporate as many informative calibrations as possible. Strategy 2 employs two joint and 16 minimum-bound calibrations modelled using a truncated Cauchy distribution that has a long tail [17]. Using minimum-bound calibrations led to old time estimates if the calibrations were not compensated by maximum bounds. Hence, calibration strategy 2 resulted in the oldest divergence time estimates. The divergence times estimated in [3] (table 1 and figure 1*c*) are based on a balanced set of minimum- and maximum-bound calibrations based on a careful examination of the fossil record, thus providing a more reliable timeline of mammal evolution than the one obtained using strategy 1 or 2. Further refinement of this timescale may be achieved by deriving many more calibrations from probabilistic estimates of clade age based on intrinsic fossil evidence [11], or the inclusion of fossils as dated-tips within molecular clock analyses [18].

The ages of placental groups presented here, together with those from recent studies [2,3], favour an early Palaeogene (i.e. post K–Pg) scenario for the diversification of placental ordinal level crown groups [2,3]. However, they also establish the origin of Placentalia firmly within the Cretaceous, supporting Archibald and Deutchman's [7] long fuse model and rejecting the explosive model of placental origination in the Palaeocene advocated by O'Leary *et al.* [8].

## Data accessibility

The trees with fossil calibrations are available as the electronic supplementary material. The genome-scale alignment is available at http://abacus.gene.ucl.ac.uk/ziheng/data.html.

## Funding statement

This work was financially supported by BBSRC grant no. BB/J009709/1.
